# Index to Predict In-hospital Mortality in Older Adults after Non-traumatic Emergency Department Intubations

**DOI:** 10.5811/westjem.2017.2.33325

**Published:** 2017-04-19

**Authors:** Kei Ouchi, Samuel Hohmann, Tadahiro Goto, Peter Ueda, Emily L. Aaronson, Daniel J. Pallin, Marcia A. Testa, James A. Tulsky, Jeremiah D. Schuur, Mara A. Schonberg

**Affiliations:** * Brigham and Women’s Hospital, Department of Emergency Medicine, Boston, Massachusetts; † Harvard Medical School, Department of Emergency Medicine, Boston, Massachusetts; ‡ Ariadne Labs, Serious Illness Care Program, Boston, Massachusetts; § Vizient, Center for Advanced Analytics, Irving, Texas; ¶ Rush University, Department of Health Systems Management, Chicago, Illinois; || Massachusetts General Hospital, Department of Emergency Medicine, Boston, Massachusetts; # Harvard T.H. Chan School of Public Health, Department of Global Health and Population, Boston, Massachusetts; ** Harvard T.H. Chan School of Public Health, Department of Biostatistics, Boston, Massachusetts; †† Dana-Farber Cancer Institute, Department of Psychosocial Oncology and Palliative Care, Boston, Massachusetts; ‡‡ Brigham and Women’s Hospital, Department of Medicine, Division of Palliative Medicine, Boston, Massachusetts; §§ Beth Israel Deaconess Medical Center, Department of Medicine, Boston, Massachusetts

## Abstract

**Introduction:**

Our goal was to develop and validate an index to predict in-hospital mortality in older adults after non-traumatic emergency department (ED) intubations.

**Methods:**

We used Vizient administrative data from hospitalizations of 22,374 adults ≥75 years who underwent non-traumatic ED intubation from 2008–2015 at nearly 300 U.S. hospitals to develop and validate an index to predict in-hospital mortality. We randomly selected one half of participants for the development cohort and one half for the validation cohort. Considering 25 potential predictors, we developed a multivariable logistic regression model using least absolute shrinkage and selection operator method to determine factors associated with in-hospital mortality. We calculated risk scores using points derived from the final model’s beta coefficients. To evaluate calibration and discrimination of the final model, we used Hosmer-Lemeshow chi-square test and receiver-operating characteristic analysis and compared mortality by risk groups in the development and validation cohorts.

**Results:**

Death during the index hospitalization occurred in 40% of cases. The final model included six variables: history of myocardial infarction, history of cerebrovascular disease, history of metastatic cancer, age, admission diagnosis of sepsis, and admission diagnosis of stroke/ intracranial hemorrhage. Those with low-risk scores (<6) had 31% risk of in-hospital mortality while those with high-risk scores (>10) had 58% risk of in-hospital mortality. The Hosmer-Lemeshow chi-square of the model was 6.47 (p=0.09), and the c-statistic was 0.62 in the validation cohort.

**Conclusion:**

The model may be useful in identifying older adults at high risk of death after ED intubation.

## INTRODUCTION

The vast majority (75%) of older adults with serious illness visit the emergency department (ED) in the last six months of their lives.^1^ Many of these patients often prioritize the quality of their life and quality of dying over simply living as long as possible and fear health states worse than death.^2^,^3^ However, a recent systematic review revealed that the majority (56%–99%) of older adults in the ED do not have advance directives available at the time of ED presentation.^4^ Even if advance care planning occurred before ED arrival, it is rarely recorded in the medical record,^5^ and patients’ values and goals may change based on changing health states necessitating emergency physicians (EP) to revisit patients’ goals.^6^

EPs often face much uncertainty about the potential benefit of advanced medical interventions in patients near the end of their lives.^7^ During the brief and time-pressured ED encounter, it is often difficult to discern which treatments are not beneficial, especially for seriously ill older adults.^8^,^9^ EPs wish to provide value-concordant care^10^ but do not feel adequately trained to discuss goals of care with patients,^11^ especially when prognosis is uncertain.^12^

Endotracheal intubation, often performed in this population, was designed to sustain life for patients in acute, reversible respiratory failure.^13^ However, large proportions of seriously ill older adults suffer poor outcomes such as death on a ventilator or chronic severe debility.^14^–^16^ Patients and caregivers providing consent in the acute setting are not well informed about the potential harm of this procedure and subsequent critical care since EPs themselves do not possess accurate prognostic information at the time of intubation. Patient-oriented decision aids have been used in the ED for a variety of conditions to facilitate shared decision-making with patients,^17^ yet none is available to help older adults near the end of life decide whether or not to be intubated or continue mechanical ventilation that was initiated prior to ED arrival.

We sought to understand factors associated with in-hospital mortality of older adults receiving non-traumatic ED intubation. Our objective was to create an index to predict in-hospital mortality in older adults intubated in the ED for indications other than trauma. By creating an index to predict in-hospital mortality, we hope to provide EPs with the necessary prognostic information to conduct a high quality, shared decision-making discussion with patients and/or their caregivers about whether or not to undergo ED intubation or continue mechanical ventilation that was already started prior to ED arrival.

## METHODS

### Study Design and Setting

This is a retrospective cohort study using patient-level administrative data from Vizient Clinical Data Base/Resource Manager™ (CDB/RM™). We obtained de-identified data regarding hospitalization after ED intubations. Vizient (formerly known as the University HealthSystem Consortium) is a consortium of more than 117 principal members (academic medical centers) and more than 300 affiliate hospitals across the United States, representing 95% of the nation’s non-profit academic medical centers. Nearly 300 of these hospitals participate in the CDB/RM™, comprised of patient-level administrative data. The mission of Vizient is to allow participating institutions to use the consortium data to accelerate organizational clinical performance.^18^ Data include patient demographics (age, sex, race, ethnicity), type of admission (elective, urgent, or emergent), procedure codes, diagnosis codes, length of stay and in-hospital mortality. Participating institutions submit all patient data monthly, and Vizient reviews each data submission for quality. Diagnoses were coded using the *International Classification of Diseases, Ninth Revision, Clinical Modifications (ICD-9-CM)*.^19^ There is no funding source or sponsors in this study. Our institutional review board.approved this study.

**Population Health Research Capsule**

What do we already know about this issue?

Emergency physicians (EP) intuitively understand the potential harm of ED intubation for older adults, but we are unable to accurately predict the in-hospital mortality for shared decision-making.

What was the research question?

Can we develop an index to risk stratify older adults for short-term mortality with the information available before intubation?

What was the major finding of the study?

We developed an index to risk stratify older adults that correctly sorts those who die from those who lived 62% of the time.

How does this improve population health?

EPs can use this information to paint the picture of potential harm of ED intubation for older adults to aid in the shared decision-making process.

### Cohort Selection

We included adults aged ≥75 years who underwent ED intubation at a CDB/RM™ participating site and had a subsequent ED-originated hospital admission between January 1, 2008, and December 31, 2015. We excluded older adults with trauma as their admission diagnosis and those with out-of-hospital cardiac arrest or intubation.

### Study Sample

We identified 22,374 individual patient records from CDB/RM™ that met our study criteria. We used Stata’s runiform command to randomly select one half of the records to be in the development cohort (n=10,789). We tested the reproducibility and calibration of our model with the remaining one half of the records, the validation cohort (n=11,585).

### Outcome

Our primary outcome of interest was in-hospital death during the index hospitalization.

### Factors of Interest

We considered four classes of variables available in CDB/RM™ as potential predictors of in-hospital mortality after ED intubation. We were interested in variables that would be available to EPs at the time of decision-making about intubation, as well as those that are available in CDB/RM™, including the following: patient demographics (sex, race, and age [categorized as: 75–79, 80–84, 85–89, and ≥90]; co-morbidity present on admission; origin of ED arrival (home, nursing home, hospice, other hospitals); and admission diagnosis determined by the EP. Using a Stata macro designed by Stagg et al,^20^ we used 13 co-morbidities included in the Charlson comorbidity index for our present-on-admission condition: history of myocardial infarction (MI); congestive heart failure (CHF); peripheral vascular disease (PVD); cerebrovascular disease (CVD); dementia; chronic obstructive lung disease (COPD); connective tissue disease (CTD); diabetes (DM); moderate-to-severe chronic kidney disease (CKD); hemiplegia/paraplegia; moderate-to-severe liver disease (LD); cancer (CA); and AIDS.^20^ We considered the location from which patients came to the ED as a predictor in our model because in prior studies older adults arriving to the ED from locations other than their home have been shown to be at higher risk of death.^21^

We considered seven admission diagnoses using the *ICD-9 CM codes*: sepsis (*ICD-9-CM* codes 038* 995.9* 785.52); gastrointestinal (GI) bleed (*ICD-9-CM* codes 578*); CHF (*ICD-9-CM* codes 428*); pneumonia (PNA) (*ICD-9-CM* codes 507* 481* 482* 483* 485* 486*); respiratory failure (*ICD-9-*CM codes 518* 786* 491*); altered mental status/seizure (*ICD-9-CM* codes 780*); and cerebrovascular accident/intracranial hemorrhage (CVA/ICH) (*ICD-9-CM* codes 430* 431* 432* 433* 434* 436* 437*). These admission diagnoses were chosen based on the top seven diagnoses by frequency in our cohort. We chose *ICD-9-CM* codes to define each of these conditions based on codes used in past studies.^22^,^23^ We grouped conditions based on our clinical judgment (e.g., combining chronic bronchitis and symptoms involving respiratory system). Admission diagnoses are typically determined by the clinician’s best assessment at the time of admission and may not be the final diagnoses of the hospitalization. We chose to include them despite this limitation because some are clinically highly correlated to such patients’ mortality (e.g., devastating CVA), and even if our index could not be used prior to intubation such information will still be helpful in discussions between clinicians and caregivers to decide whether to continue the aggressive medical interventions. We chose not to include socioeconomic variables (e.g., income, insurance status) in the development of our model since such variables may not be readily available to the clinician prior to ED intubation.

### Statistical Analysis

We used multivariable logistic regression using the least absolute shrinkage and selection operator (LASSO) method 24 to develop our model. This model selection technique is attractive for prognostic model building because of its ability to shrink large regression coefficients to reduce overfitting (by forcing less important variable coefficients to zero); it then automatically performs variable selection with fewer predictor variables. It has been considered by some to be superior to conventional methods (e.g. stepwise selection).^25^ To improve the clinical utility of the final model, we chose to remove variables not significantly predictive of (change in AUC>0.0035) or associated with (p<0.05) in-hospital mortality after ED intubation. We used Stata version 14.1 (StataCorp, Texas, U.S.A.) with a LASSO macro designed by Mander.^26^

To determine an individual’s mortality risk, we developed a point-based risk scoring system using methods similar to other studies.^27^ Points were assigned to each risk factor in the final model by dividing each beta coefficient by the lowest beta coefficient in the final multivariable model and then rounding to the nearest tenth decimal point. We then assigned a risk score to each individual in the development and validation cohorts by summing the points for each risk factor present for that individual. We stratified the scores into three risk groups similar to prior studies^28^,^29^ and based on our clinical judgment: low-(<6 points, 31% mortality), medium- (6 to 10 points, 40% mortality), and high-risk groups (>10 points, 58% mortality) for each cohort, and we calculated in-hospital mortality.

We assessed model calibration by examining the relationship between the expected and observed mortality for the high-risk group since we were most interested in correctly identifying the highest risk group, which is critical information to be communicated to the patient and/or their surrogates (e.g. futility of care). We tested model calibration (the ability of the model’s estimated risk to agree with actual outcomes within groups of subjects in similar predicted risk) with Hosmer-Lemeshow goodness-of-fit test using quintile of risk stratification. We used quintile of in-hospital mortality risk (calculated the risk of death and categorized the patients into five equal groups based on their risk of death) to highlight the low- and high-risk groups. We chose to use quintiles of risk based on the distribution of risk within our cohort and prior similar study.^27^ To assess calibration, we used “lfit” post-estimation command after regression modeling on Stata version 14.1 (StataCorp, Texas, U.S.A.) to compare the “expected” number of in-hospital deaths based on our model’s estimates to the “observed” number in our cohort. We assessed these comparisons within quintiles of in-hospital mortality risks (Table 3). To assess model discrimination (the ability of the model to correctly identify those who died from those who survived) we calculated a c-statistic.

## RESULTS

Of the 10,789 participants in the development cohort, 46% were male and 65% were non-Hispanic White. Overall 49% had a CCI ≥3, and 40% of participants died during the index hospitalization. The characteristics of the development and validation cohorts were similar (Table 1).

The model included one demographic variable (age group), three co-morbidity variables (MI, CVD, and metastatic CA), and two admission diagnosis variables (sepsis and stroke/intracranial hemorrhage). All variables that did not meet predictive significance (change in AUC>0.0035) or statistical significance (p<0.05) were removed during the model-building process, including the ED arrival location and some of the CCI variables. Table 2 depicts the adjusted odds ratio for in-hospital mortality from the model and the points assigned to each factor.

Our model correctly sorted patients who died from patients who lived 62% of the time in both derivation and validation cohorts (c-statistic of 0.62). Further, our model demonstrated excellent calibration (Hosmer-Lemeshow chi-square = 6.47/p=0.09) with virtually identical mortality rates in the development and validation cohorts for all predicted risk groups (Table 3). Of the 1,106 participants predicted to die in the highest risk quintile of validation cohort, 1,096 participants actually died (>99.1%). In-hospital mortality ranged from 30% in the lowest-risk quintile to 57% in the highest-risk quintile in the development cohort and from 30% in the lowest-risk quintile to 57% in the highest-risk quintile in the validation cohort.

## DISCUSSION

By using CDB/RM™, we developed and validated an index to predict in-hospital mortality for U.S. adults ≥75 years receiving non-traumatic ED intubation. Our rule demonstrated excellent calibration with minimal under-/over-estimation of risk within our cohort for the highest risk score group (926 expected death and 910 observed death, <3% difference) and fair predictive ability (correctly sorted patients who died from patients who lived 62% of the time) as demonstrated by increasing risk of in-hospital mortality by point score. Older adults in the highest risk group (>10 points) had 58% (range 56–84%) probability of in-hospital mortality. After validation in the clinical settings, our simple index may be a valuable tool for EPs to identify older adults at high risk of in-hospital mortality after an ED intubation.

ED intubation for older adults is a life-changing event. The most common reasons for acute respiratory failure in older adults are CHF (43%), PNA (35%), and COPD (32%),^30^ and they are associated with high in-hospital mortality (>20%,^31^ 53%,^32^ and 40%^33^ respectively.). Among the survivors, as many as 13% will require prolonged mechanical ventilation (defined by ≥21 days for ≥6 hours per day).^34^ In older adults, 35% will never meet the criteria for weaning from the ventilator at this stage and have 65% probability of dying in the long-term care facility, with median survival ranging from 2.1 to 4.4 months.^35^ Even if successfully weaned from the ventilator, 40% will sustain severe functional disability after the hospital discharge unless the baseline functional status is completely normal.^36^ The degree of potential harm from continuing critical care after ED intubation is clearly not well communicated to older adults since 74% of older adults would not choose treatment if the burden of treatment were high and the anticipated survival were to come with severe functional impairment.^37^ Further, >50% of older adults consider “rely[ing] on a breathing machine to live” worse than death.^3^

The first step to informing older adults about the potential harm of ED intubation and subsequent critical care is discussing the probability of in-hospital mortality. Our hope is that this index will allow EPs to accurately describe to older adults and their caregivers the potential for harm. With the prediction of in-hospital mortality, EPs can better facilitate the shared decision-making process to provide care concordant with patient/caregiver’s goals. It may help older adults unlikely to benefit from ED intubation and ongoing critical care to avoid medical treatment that is not going to prolong their lives and instead may jeopardize their chance of a peaceful death.

Despite seeing many critically ill seniors, EPs often face prognostic uncertainty when providing care to seriously ill older adults. There is also a great deal of uncertainty concerning which medical procedures are likely to help these seriously ill elders versus those that are only going to cause harm.^38^ Previous studies suggested the grim prognosis (30.2% in-hospital mortality for non-traumatic patients with average age of 65) of older adults intubated in the ED,^16^ but the information to risk stratify them based on available information was limited. To our knowledge, this investigation provides the first evidence to inform the probability of in-hospital mortality in older adults intubated in the ED for indications other than trauma. We carefully selected each potential predictor based on the type of information likely to be available before the decision to intubate.

Although we demonstrated that our index is likely to identify high-risk patients (58% of in-hospital mortality in the highest risk group), the threshold of futile intervention will vary for different patients. Thus, some older adults or clinicians may require a much higher level of certainty in risk (>99% risk of in-hospital mortality) in order to refrain from ED intubation and ongoing critical care – a medical procedure developed to help patients avoid death. Therefore, once our index is externally validated, an effort should be made to shape how this evidence is presented to the patients and their caregivers. Now that we have an index to accurately identify high-risk patients, the most effective method to communicate this information must be investigated to enhance the shared decision-making process.^39^

## LIMITATIONS

Our investigation has notable limitations. First, our index was developed using administrative data, which are subject to standard limitations including accuracy of data received (e.g., dementia is under-diagnosed) and limited clinical information (e.g. physiological measurements and laboratory data).^40^,^41^ Since some clinical parameters such as pre-ED frailty can be a strong predictor of mortality after critical illness,^42^ the lack of such information in our database posed a major limitation. Second, we were unable to include the individuals for whom intubation was considered but not performed in this cohort because such individuals could not be identified using our database. Although they are not included, these individuals likely have much higher mortality and are beyond the scope of our study.

Third, we were unable to include pre-existing do-not-resuscitate orders or caregiver’s stated preferences of the patient in the ED as part of our index due to our administrative data source. Fourth, we were unable to assess whether our index performs comparatively to the clinician’s overall clinical assessment (i.e., clinician gestalt) due to our data source limitation. Fifth, the hospitals that participate in Vizient CDB/RM™ are disproportionally academic and may not be representative of all U.S. hospitals. Sixth, the index has yet to be validated in a clinical setting. Seventh, we were unable to exclude patients who had surgery during the index hospitalization. Such a subpopulation may have had a higher mortality at baseline compared to all others within our cohort. Finally, the difference in predicted mortality from 31% in low-risk patients to 58% in high-risk patients may or may not alter an EP’s decision to intubate. Rather, we hope that such information is useful for EPs to facilitate the shared decision-making discussions with the patients and caregivers.

## CONCLUSION

In summary, we have developed an index to predict in-hospital mortality in older adults intubated in the ED for indications other than trauma. Patients with a score >10 had a 58% (range 56–83%) probability of in-hospital mortality. This index can provide useful information for EPs to discuss the potential harm/benefit of ED intubation and continuing mechanical ventilation with older adults and their caregivers to provide care concordant with their values and preferences.

## Figures and Tables

**Table 1:** Demographic and potential predictors of the development and validation cohorts and unadjusted mortality odds ratios in a study assessing the feasibility of creating an index to risk stratify older adults for in-hospital mortality after intubation.

	Development cohort (n =10,789)		Validation cohort (n = 11,585)	
		
Category	Weighted %	Unadjusted OR (95% CI)	Weighted %	Unadjusted OR (95% CI)
Age				
75 – 79 Years	34%	Reference	34%	Reference
80 – 84 Years	31%	1.28 (1.16–1.41)	30%	1.32 (1.20–1.45)
85 – 89 Years	23%	1.48 (1.33–1.64)	23%	1.44 (1.30–1.59)
>= 90 Years	12%	1.74 (1.54–1.98)	13%	2.05 (1.81–2.31)
Sex				
Men	46%	Reference	45%	Reference
Women	54%	1.01 (0.94–1.10)	51%	0.99 (0.92–1.07)
Race				
White	65%	Reference	66%	Reference
Black	21%	0.71 (0.65–0.79)	22%	0.81 (0.70–0.92)
Others	14%	1.00 (0.92–1.08)	12%	1.06 (0.96–1.18)
Comorbid conditions				
History of myocardial infarction	14%	1.19 (1.07–1.32)	14%	1.26 (1.14–1.40)
Congestive heart failure	42%	0.71 (0.65–0.77)	42%	0.69 (0.64–0.74)
Peripheral vascular disease	10%	1.15 (1.01–1.32)	10%	1.23 (1.09–1.40)
Dementia	3%	0.71 (0.55–0.90)	3%	0.72 (0.57–0.91)
Cerebrovascular accident	21%	1.80 (1.64–1.97)	22%	1.77 (1.62–1.93)
Chronic obstructive lung disease	33%	0.63 (0.58–0.68)	33%	0.65 (0.60–0.70)
Connective tissue disease	3%	1.00 (0.79–1.27)	3%	1.18 (0.95–1.45)
Diabetes	34%	0.85 (0.78–0.92)	33%	0.82 (0.75–0.88)
Chronic kidney disease	31%	0.88 (0.81–0.96)	30%	0.86 (0.79–0.94)
Hemiplegia/paraplegia	6%	1.21 (1.10–1.34)	6%	1.31 (1.14–1.51)
Moderate liver disease	1%	1.68 (1.15–2.45)	1%	1.49 (1.04–2.14)
Metastatic cancer	4%	2.10 (1.68–2.55)	3%	1.71 (1.40–2.10)
Origin of ED arrival				
Home	75%	Reference	74%	Reference
Nursing home	7%	0.94 (0.84–1.05)	7%	0.84 (0.72–1.44)
Hospice	0.02%	0.92 (0.17–5.09)	0.03%	6.18 (0.63–60.53)
Other hospitals	24%	1.10 (0.87–1.40)	24%	1.12 (1.03–1.22)
Admitting diagnosis				
Sepsis	11%	1.38 (1.21–1.56)	11%	1.41 (1.25–1.60)
Gastrointestinal bleed	1%	1.15 (0.91–1.44)	1%	0.83 (0.58–1.18)
Congestive heart failure	1%	0.78 (0.55–1.10)	2%	0.52 (0.36–0.74)
Pneumonia	4%	0.83 (0.71–0.97)	3%	1.08 (0.88–1.33)
Respiratory failure	31%	0.56 (0.52–0.59)	32%	0.52 (0.48–0.57)
Altered mental status	15%	0.72 (0.67–0.78)	15%	0.71 (0.64–0.79)
Cerebrovascular accident/intracranial hemorrhage	10%	2.4 (2.17–2.58)	10%	2.34 (2.05–2.66)

*OR*, odds ratio; *CI*, confidence interval.

**Table 2:** Adjusted beta coefficients/odds ratios and points assigned to each risk factor for older adults intubated in the emergency department.

Risk factor	Beta coefficient (95% CI)	Odds ratio (95% CI)	Points
Co-morbid condition			
Myocardial infarction	0.284 (0.162–0.105)	1.33 (1.18 – 1.50)	1.2
Cerebral vascular disease	0.33 (0.20 – 0.462)	1.40 (1.22 – 1.59)	1.4
Metastatic cancer	0.923 (0.691–1.155)	2.52 (2.00 – 3.18)	4
Age			
75 – 79 Years	Reference	Reference	0
80 – 84 Years	0.23 (0.122 – 0.338)	1.26 (1.13 – 1.4)	1
85 – 89 Years	0.4 (0.280 – 0.511)	1.48 (1.32 – 1.67)	1.7
≥90 Years	0.6 (0.456 – 0.737)	1.82 (1.59 – 2.10)	2.6
Admission diagnosis			
Sepsis	0.441 (0.309 – 0.572)	1.55 (1.36 – 1.77)	1.9
Stroke/intracranial hemorrhage	0.723 (0.547–0.899)	2.10 (1.73 – 2.46)	3.1
Total possible points			12.3

*CI*, confidence interval.

**Table 3:** In-hospital mortality in the development and validation cohorts.

	Development		Validation		Death	
			
Quintile of risk	n	Mortality (range)	N	Mortality (range)	Observed	Expected
1	3175	30% (28 – 33%)	3484	30% (28 – 33%)	1014	1056
2	619	35% (34 – 35%)	626	35% (34 – 35%)	236	217
3	1919	38% (37 – 41%)	2096	38% (37 – 41%)	785	793
4	1619	43% (42 – 47%)	1729	43% (41 – 47%)	771	749
5	1833	57% (48 – 81%)	1958	57% (48 – 84%)	1096	1106
Point score						
Low (<6)	3175	31% (23 – 33%)	3484	31% (28 – 33%)	1014	1055
Medium (6–10)	4489	40% (38 – 55%)	4817	40% (40 – 55%)	2492	2456
High (>10)	1501	59% (51 – 81%)	1592	58% (56 – 84%)	910	926
